# Single-Nucleotide Polymorphism (SNP) A986S (rs1801725) of the Calcium-Sensing Receptor in Children With Idiopathic Hypercalciuria

**DOI:** 10.7759/cureus.105619

**Published:** 2026-03-21

**Authors:** Madhura N S, Shivalingaiah M, Kowsalya R

**Affiliations:** 1 Biochemistry, Institute of Nephro Urology, Bengaluru, IND; 2 Urology, Institute of Nephro Urology, Bengaluru, IND

**Keywords:** a986s polymorphism, calcium-sensing receptor, casr gene, idiopathic hypercalciuria, pediatric nephrolithiasis, rs1801725

## Abstract

Background

Idiopathic hypercalciuria (IH) is a common metabolic abnormality in children and an important risk factor for hematuria, nephrolithiasis, and recurrent urinary symptoms. Genetic factors, particularly variations in the calcium-sensing receptor (CaSR) gene, may predispose individuals to altered calcium homeostasis. The CaSR A986S polymorphism (rs1801725) has been implicated in abnormal calcium metabolism and stone disease, but evidence in pediatric populations remains limited.

Objective

To investigate the association between the CaSR A986S (rs1801725) polymorphism and IH in children.

Methods

A case-control study was conducted at a tertiary nephrology center in Bangalore between January 2022 and May 2023. Sixty children aged 4-14 years with IH were compared with 60 age- and sex-matched controls. Clinical characteristics, serum calcium levels, and 24-hour urinary calcium excretion were assessed. Genotyping for rs1801725 (A986S) was performed, and genotype frequencies, p-values, and ORs were calculated using SPSS software.

Results

Children with IH had significantly higher 24-hour urinary calcium excretion than controls (413 ± 76 vs 215 ± 132 mg/day; p = 0.001). The mutant TT genotype was more frequent in patients with IH than in controls (15% vs 1.7%; p = 0.02), while the normal GG genotype was significantly less common in patients with IH (60% vs 91%; p = 0.0002). Allelic analysis showed a significantly higher frequency of the T allele in patients with IH (35% vs 5%), conferring a markedly increased risk of IH (OR = 10.23; 95% CI: 2.85-36.66; p = 0.0004).

Conclusion

The CaSR A986S (rs1801725) polymorphism is significantly associated with IH in children. The T allele, particularly the TT and GT genotypes, confers a markedly increased risk of IH, suggesting a meaningful genetic contribution to calcium dysregulation in the pediatric population. These findings support the potential role of CaSR genotyping in early risk stratification and individualized management of IH.

## Introduction

Idiopathic hypercalciuria (IH) is one of the most common metabolic and urological disorders in children, presenting mainly with hematuria, UTI, and kidney stone formation. It is characterized by excessive calcium excretion in the urine without any identifiable metabolic or endocrine disorder [1-2]. Children with IH usually have normal blood calcium and parathyroid hormone levels, distinguishing it from secondary causes of hypercalciuria [3]. The prevalence of IH in children is estimated to be 3-6% in the general pediatric population and 30-60% among those with urolithiasis or recurrent hematuria [4]. The pathophysiology of the disease is multifactorial and is influenced by environmental and genetic factors. A familial tendency is well documented, with an autosomal dominant inheritance pattern [5-7].

The calcium-sensing receptor (CaSR), located on chromosome 3q13.3-21, plays an important role in maintaining calcium homeostasis by regulating calcium reabsorption in the renal tubules and controlling parathyroid hormone secretion [8-12]. Genetic variations in the CaSR gene could alter CaSR function, leading to abnormal calcium reabsorption in the renal tubules and contributing to the pathogenesis of IH [11-13]. Single-nucleotide polymorphisms (SNPs) in the CaSR gene have been implicated in altered calcium metabolism. Among them, the A986S variant (rs1801725, G→T, Ala→Ser substitution) has been widely studied. This SNP has been associated with higher serum calcium concentrations, increased urinary calcium excretion, and a greater risk of nephrolithiasis in adults [12-14]. Although data in children are limited, recent studies suggest that the CaSR A986S (rs1801725) polymorphism may increase susceptibility to IH in pediatric patients. Identification of such genetic variants may help explain inter-individual variability in calcium excretion and could contribute to improved risk stratification and early management of affected children. Understanding this association could help in better risk assessment and individualized management of IH in children. In the present study, we aimed to investigate the association between the CaSR A986S (rs1801725) polymorphism and IH in children.

## Materials and methods

This was a single-centre, observational case-control study conducted at the Institute of Nephro-Urology, Bangalore, a tertiary referral centre for nephrology and urology in Karnataka, between January 2022 and May 2023. Ethical clearance for the study was obtained from the Institutional Ethics Committee, and written informed consent was obtained from the parents or legal guardians of all participating children.

Cases

Children aged 4-14 years, identified through biochemical screening in the laboratory, with idiopathic hypercalciuria diagnosed based on the following criteria were included: (a) 24-hour urinary calcium >4 mg/kg/day and (b) normal serum levels of calcium, phosphate, and parathyroid hormone. Such participants were included after obtaining informed consent from their parents or guardians.

Controls 

Age- and sex-matched individuals without a history of urolithiasis, with normal urinary calcium excretion, and without a family history of urolithiasis were enrolled.

Children with primary hyperparathyroidism, renal tubular acidosis, chronic kidney disease, or metabolic disorders affecting calcium metabolism were excluded from the study. Children with known genetic syndromes, congenital renal anomalies, and active UTI were also excluded.

Sample collection and storage

Blood samples were collected under aseptic precautions using standard venipuncture into plain and ethylenediaminetetraacetic acid (EDTA) tubes. Samples in EDTA tubes were stored at -20°C until DNA extraction and were used for genotyping. Samples in plain tubes were allowed to clot completely, then centrifuged for 15 minutes. The serum was separated and analyzed on the same day for biochemical tests [15].

For 24-hour urine collection, all participants were given a container and were instructed to discard the first morning urine, note the time, and collect all urine for the next 24 hours. The final sample was collected at the same time the following day. The collected urine was then submitted to the laboratory on the same day, as instructed.

DNA extraction 

Genomic DNA was extracted from peripheral blood using the QIAamp DNA Blood Mini Kit (Catalogue No. 51104, Qiagen GmbH, Hilden, Germany) in the RT-PCR laboratory of our institute. The extraction was based on the silica membrane spin-column method and involved four main steps: (1) lysis of blood cells, (2) binding of DNA to the silica column, (3) washing to remove contaminants, and (4) elution to obtain purified DNA. The extracted DNA was stored at -20°C until genotyping.

Genotyping 

SNP genotyping was performed using the hydrolysis probe method. The TaqMan® SNP Genotyping Assay (Applied Biosystems, USA) was used to analyze rs1801725 of the CASR gene on the QIAquant 96 5plex real-time qPCR system (Qiagen). This method uses the 5′ nuclease activity of Taq polymerase to produce a fluorescent signal during PCR. For SNP rs1801725, two probes were used: one for the wild-type allele and one for the variant allele. Each probe was labeled with a reporter dye (FAM or VIC) at the 5′ end and a quencher dye (MGB/BHQ) at the 3′ end. Fluorescence occurs only when the probe binds perfectly to the target sequence. The ratio of the two fluorescent signals was measured to determine the genotype of each sample.

Biochemical analysis 

Serum biochemical analyses were performed using the Abbott Alinity™ chemistry and immunoassay system, comprising the Alinity c and Alinity i analyzers (Abbott Laboratories, Abbott Park, IL, USA), in the Biochemistry laboratory. Ready-to-use kits from the Abbott Alinity c and i systems were utilized for the analyses. Total serum calcium was measured by the Arsenazo III dye-binding method, and serum 25-hydroxyvitamin D (25-OH D) levels were measured using chemiluminescent immunoassay (CLIA).

Statistical analysis

Data were analyzed using appropriate statistical methods. Continuous variables were expressed as mean ± SD, and categorical variables were expressed as frequencies and percentages. Comparison of continuous variables between IH patients and controls was performed using Student’s t-test. Categorical variables, including sex distribution and genotype frequencies, were compared using the chi-square (χ²) test. Allele frequencies were calculated by direct counting. The strength of association between the rs1801725 polymorphism and idiopathic hypercalciuria was estimated by calculating the OR with a 95% CI. A p-value < 0.05 was considered statistically significant.

Sample size calculation

Sample size was calculated assuming the following: minor allele frequency (A986S T allele), ~15-20%; expected OR, 1.8-2.0; power, 80%; α, 0.05; and case:control ratio, 1:1. This gave an approximate sample size of 60-65 cases and 60-65 controls.

## Results

Among the 60 children with IH, clinical presentations were heterogeneous and overlapping. Hematuria was the most common isolated symptom, observed in 10 children (16.7%), followed by abdominal/flank pain in 7 (11.7%) and renal stones in 6 (10.0%). Several children presented with combined symptoms, most commonly hematuria with renal stones (13.3%), followed by hematuria with abdominal/flank pain (10.0%) and renal stones with abdominal/flank pain (8.3%). A smaller proportion presented with hematuria with dysuria (6.7%) and recurrent urinary tract infection associated with hematuria and/or stones (5.0%).

Eighteen children (30.0%) were asymptomatic at presentation and were diagnosed incidentally during evaluation (Table 1).

**Table 1:** Clinical presentations of children with idiopathic hypercalciuria.

Symptom combination	Number of children, n (%)
Hematuria only	10 (16.7%)
Renal stones only	6 (10.0%)
Abdominal/flank pain only	7 (11.7%)
Hematuria + renal stones	8 (13.3%)
Hematuria + abdominal/flank pain	6 (10.0%)
Renal stones + abdominal/flank pain	5 (8.3%)
Hematuria + dysuria	4 (6.7%)
Recurrent UTI with hematuria and/or stones	3 (5.0%)
Asymptomatic	18 (30.0%)

Table 2 compares the demographic and biochemical characteristics of children with IH and age-matched healthy controls (n = 60 in each group). The mean age of patients with IH was 8 ± 3 years, which was comparable to 7 ± 4 years in controls (p = 0.124). The male-to-female ratio was similar in both groups (38:22 vs 36:24; p = 0.635), indicating no statistically significant sex difference. Serum calcium levels were within the normal reference range in both groups (IH: 9.7 ± 1.2 mg/dL; controls: 10.0 ± 0.9 mg/dL; p = 0.12). However, 24-hour urinary calcium excretion was significantly higher in the IH group (413 ± 76 mg/day) than in controls (215 ± 132 mg/day; p = 0.001), which is consistent with the diagnostic hallmark of hypercalciuria.

**Table 2:** Characteristics of patients with IH and controls. Continuous variables were analyzed using Student’s t-test, and categorical variables were analyzed using the chi-square test. IH: Idiopathic hypercalciuria.

Characteristics	IH patients (n = 60)	Controls (n = 60)	P-value
Age (years)	8 ± 3	7 ± 4	0.124
Male, n (%)	38 (63.3%)	36 (60.0%)	0.635
Female, n (%)	22 (36.7%)	24 (40.0%)	
Serum calcium (8.4-10.2 mg/dL)	9.7 ± 1.2	10.0 ± 0.9	0.12
24-hour urinary calcium (100-300 mg/day)	413 ± 76	215 ± 132	0.001

The genotype distribution for rs1801725 is detailed in Table 3. The mutant TT genotype of the rs1801725 polymorphism was markedly more frequent among IH patients than controls (15% vs 3%; p = 0.02). The normal GG genotype was significantly less frequent in IH patients (60%) than in controls (91.6%) (p = 0.0002), while the GT genotype (25% vs 6.6%; p = 0.008) was also significantly more frequent in IH patients than in controls.

**Table 3:** Genotype distribution of SNP rs1801725. Genotype frequencies between patients with IH and controls were compared using the chi-square (χ²) test. A p-value < 0.05 was considered statistically significant. IH: Idiopathic hypercalciuria; SNP: Single nucleotide polymorphism.

Genotypes	IH, n (%) (n = 60)	Controls, n (%) (n = 60)	P-value
GG	36 (60.0%)	55 (91.7%)	0.0002
GT	15 (25.0%)	4 (6.7%)	0.008
TT	9 (15.0%)	1 (1.7%)	0.02

Table 4 shows the allelic distribution of SNP rs1801725 among individuals with IH (n = 60) and controls (n = 60). The mutant T allele was present in 35% (21/60) of IH cases, whereas it was observed in only 5% (3/60) of the control group. The calculated odds ratio (OR = 10.23; 95% CI: 2.85-36.66) suggests that individuals carrying the T allele have more than a 10-fold increased risk of developing IH compared with others. The association was found to be statistically highly significant (p = 0.0004).

**Table 4:** Allelic distribution of SNP rs1801725. Allelic frequencies were compared using the chi-square test. The OR with 95% CI was calculated to assess the risk association. IH: Idiopathic hypercalciuria; SNP: Single nucleotide polymorphism.

SNP ID	Allele	IH, n (%) (n = 60)	Controls, n (%) (n = 60)	OR (95% CI)	P-value
rs1801725	T	21 (35.0%)	3 (5.0%)	10.23 (2.85 to 36.66)	0.0004
	G	39 (65.0%)	57 (95.0%)		

## Discussion

IH is the most common metabolic abnormality associated with nephrolithiasis in children and is defined as increased urinary calcium excretion in the absence of hypercalcemia [1-3]. The pathophysiology of IH is heterogeneous, involving increased intestinal calcium absorption, impaired renal tubular calcium reabsorption, enhanced bone resorption, or a combination of these mechanisms [4-5]. Some children may exhibit absorptive hypercalciuria due to increased intestinal calcium uptake, while others demonstrate renal hypercalciuria resulting from decreased tubular calcium reabsorption. In certain cases, increased bone turnover contributes to elevated urinary calcium excretion [6-7].

Genetic predisposition plays a significant role in IH. Familial clustering and autosomal dominant inheritance patterns have been described, suggesting that variations in genes regulating calcium transport and sensing contribute to disease susceptibility. Among these, the CaSR is of particular importance, as it regulates parathyroid hormone secretion and renal calcium handling [8-10].

Genetic polymorphisms in CaSR may alter receptor sensitivity to extracellular calcium, leading to inappropriate renal calcium excretion despite normocalcemia. This supports the hypothesis that IH may represent a genetically mediated defect in calcium sensing [8-13].

In a study conducted by Vezzoli G et al. (2002), individuals carrying the TT genotype of the CaSR A986S polymorphism (rs1801725) demonstrated a significantly increased risk of hypercalciuria and higher urinary calcium excretion compared with other genotypes [8]. However, this association was established in an adult population, and therefore its applicability to pediatric patients remains to be clearly determined.

In the present study, we evaluated the association between the CaSR A986S (rs1801725) polymorphism and IH in children. Our findings demonstrated a significantly higher frequency of the GT and TT genotypes among IH patients compared with controls. In addition, the mutant T allele of rs1801725 was significantly more frequent among children with IH than among controls, with a markedly elevated odds ratio (OR = 10.23), indicating a strong association between the T allele and susceptibility to IH.

Our findings could be explained by the mechanisms described by Vezzoli G et al. [11], which highlight the role of CaSR gene variants in calcium homeostasis and stone formation. They concluded in their study that the CaSR gene plays an important role in regulating urinary calcium excretion and that its polymorphisms may predispose individuals to hypercalciuria and stone formation. In another study, the authors concluded that there is a significant genetic association between the CaSR gene polymorphism and the risk of developing calcium urolithiasis in the Russian population [12].

Our findings demonstrating a significant association between rs1801725 (A986S) and IH are supported by several previous studies across different populations. A recent Indian study reported an association between CaSR polymorphisms and both the occurrence and recurrence of nephrolithiasis [13]. Likewise, in a study by Hou J et al., the rs1801725 GT genotype was found to be significantly associated with an increased risk of urinary calcium stone formation and higher 24-hour urinary calcium excretion in the Chinese population [14]. Further, Ferreira LG et al. (2010) highlighted the combined influence of vitamin D receptor and CaSR gene polymorphisms in hypercalciuric stone formers [16]. In addition, Hamilton DC et al. (2009) demonstrated that deviations from Hardy-Weinberg equilibrium in case groups may reflect underlying genetic heterogeneity in renal stone disease, supporting the concept that CaSR polymorphisms contribute as susceptibility factors rather than monogenic causes [17].

The present study provides preliminary evidence from the pediatric population suggesting that CaSR polymorphisms may be associated with susceptibility to IH in children. Identification of such genetic determinants may have potential implications for risk stratification, early detection, and individualized management of children at risk for nephrolithiasis or recurrent hematuria. However, these findings require confirmation in larger multicenter studies involving diverse pediatric populations to better establish the role of CaSR variants in IH.

Limitations 

The present study has certain limitations. First, the relatively small sample size and single-center design may limit the generalizability of the findings to the broader population. Second, functional analysis of the rs1801725 polymorphism was not performed; therefore, the exact biological mechanism underlying the observed association could not be established. Third, lifestyle factors such as dietary calcium intake, sodium consumption, and hydration status, which significantly influence urinary calcium excretion, were not systematically assessed. Larger multicenter studies with diverse pediatric populations are required to validate these findings and improve generalizability.

## Conclusions

In conclusion, children with IH demonstrated significantly higher urinary calcium excretion and a higher frequency of the CaSR A986S (rs1801725) T allele compared with controls, suggesting a possible genetic contribution to disease susceptibility. These findings suggest a potential role for CaSR polymorphisms in the genetic susceptibility to IH in the pediatric population. Identification of such genetic variants is clinically important, as it may facilitate early risk assessment, enable personalized management strategies, and help prevent complications such as nephrolithiasis and recurrent hematuria in susceptible children. Incorporating genetic insights into clinical evaluation could improve early detection and guide targeted interventions in children predisposed to IH. Future multicentric studies with larger cohorts are warranted to validate these findings and further clarify the role of CaSR variants in pediatric hypercalciuria.
